# Diagnostic accuracy of combined optical- and radio-guided SNB for neck staging of oral squamous cell carcinoma lesions in the anterior oral cavity

**DOI:** 10.1007/s00405-023-07939-5

**Published:** 2023-04-03

**Authors:** Anders Christensen, Irene Wessel, Birgitte Wittenborg Charabi, Karina Juhl, Katalin Kiss, Giedrius Lelkaitis, Jann Mortensen, Andreas Kjaer, Christian von Buchwald, Jesper Filtenborg Tvedskov

**Affiliations:** 1 Department of Otolaryngology, Head & Neck Surgery and Audiology, 6033, Copenhagen University Hospital, Rigshospitalet, Blegdamsvej 9, 2100 Copenhagen, Denmark; 2 Department of Clinical Physiology and Nuclear Medicine & Cluster for Molecular Imaging & Department of Biomedical Sciences, Copenhagen University Hospital, Rigshospitalet, University of Copenhagen, Copenhagen, Denmark; 3 Department of Pathology, Copenhagen University Hospital, Rigshospitalet, Copenhagen, Denmark

**Keywords:** Indocyanine green, Near-infrared optical imaging, Sentinel node biopsy, Oral squamous cell carcinoma, Neck metastasis

## Abstract

**Purpose:**

The purpose was to investigate the diagnostic performance of bimodal optical and radio-guided sentinel node biopsy (SNB) for oral squamous cell carcinoma (OSCC) sub-sites in the anterior oral cavity.

**Methods:**

Prospective study of 50 consecutive patients with cN0 OSCC scheduled for SNB was injected with the tracer complex Tc99m:ICG:Nacocoll. A near-infrared camera was applied for optical SN detection. Endpoints were modality for intraoperative SN detection and false omission rate at follow-up.

**Results:**

In all patients, a SN could be detected. In 12/50 (24%) of cases, the SPECT/CT showed no focus in level 1, but intraoperatively a SN in level 1 was optically detected. In 22/50 cases (44%), an additional SN was identified only due to the optical imaging. At follow-up, the false omission rate was 0%.

**Conclusion:**

Optical imaging appears to be an effective tool to allow real-time SN identification comprising level 1 unaffected by possible interference of radiation site from the injection.

## Background

In early-stage oral cavity cancer with a clinically negative neck (cN0), the risk of subclinical nodal neck disease is 20–30% [1]. Selective neck dissection has traditionally been performed to manage the cN0, with a dual purpose of neck staging and treatment of possible occult metastatic disease [2]. However, with this approach, up to 80% of the patients, with a pN0 neck, will be over-treated with a unilateral or bilateral neck dissection and possible morbidity related to this procedure. As an alternative to neck dissection, sentinel node biopsy (SNB) has gradually been validated and implemented during the last two decades as a staging method to select those patients that harbor occult neck metastasis and will benefit from a neck dissection [3]. In addition, SNB enables detection of bilateral or contralateral lymphatic drainage patterns in the neck or drainage to lymph nodes located in neck levels outside levels I–III, which are typically included in a selective neck dissection for cN0 oral cancer [4]. Recently, two prospective randomized controlled trials comparing SND and SNB showed oncological non-inferiority and a more favorable morbidity profile of SNB [5, 6]. However, despite overall acceptable diagnostic performance of SNB neck staging across all tumor sub-sites, higher false negative rates have been reported for tumors located in the anterior oral cavity and especially in floor of the mouth (FOM), which has been an issue of concern [7, 8]. The reason for a lower accuracy of SNB to stage the neck in FOM tumors is most likely the shine-through phenomenon. When a SN with subclinical metastatic disease is located in close proximity to the primary injection site in level 1, it may be overshadowed by radiation from the injection site, and thus remains undetected on preoperative imaging as well as intraoperatively. Consequently, as stated in the SNB consensus guideline from 2019, SNB neck staging of FOM tumors should be carried out in conjunction with a super-selective level 1 neck dissection (ND) or by the use of an additional tracer to allow intraoperative SN detection based on a non-radioactive signal, for example an optical dye [9]. The concept and the technique of super-selective level 1 ND in conjunction with SNB, to ensure accurate neck staging of FOM tumors, have been described by Stoeckli and colleges [10]. To address the challenge of shine-through, optical imaging alone or in conjunction with a radio-tracer has recently been explored [11–14]. By the use of an optical tracer, data indicates that intraoperative guidance toward a SN, independent from radioactivity guidance, is feasible [14, 15]. Encouraging results have been reported showing high SN identification rates using optical technique. Also, optical imaging had the ability to detect SNs that were not visualized on radio-tracer imaging [16–18]. However, data on possible impact of additional use of optical guidance for SNB neck staging in OSCC on false negative rates deducted from follow-up data has not been reported.

Hence, the purpose of this study was to investigate the diagnostic performance of bimodal optical and radio-guided SNB to stage the cN0 neck in early OSCC for tumors localized in the anterior oral cavity. Endpoints were modality for intraoperative SN detection (optical vs radioactive) and false omission rate of SNB based on N-site failures at follow-up.

## Methods

### Patients

A prospective single-arm clinical study was conducted at Copenhagen University Hospital-Rigshospitalet, a tertiary cancer center in Denmark, between November 2017 and December 2020. Consecutive patients with primary early-stage cT1-T2N0M0 OSCC, scheduled for SNB staging were enrolled. Only patients with biopsy-verified squamous cell carcinoma located in the anterior oral cavity with the following sub-sites were included: FOM, inferior surface of tongue, buccal mucosa, and lower gum. These sub-sites were selected due to a possible higher reported risk of false negative results of SNB caused by the shine-through phenomenon. The nodal neck status prior to surgery was evaluated with clinical examination, ultrasonography, MRI, and/or CT. Exclusion criteria were previous head and neck cancer, previous, radiotherapy or surgery to the neck, and pregnancy or allergy to ICG or iodine. From all patients, a written informed consent to participate was obtained. The study was approved by the Danish Regional Scientific Ethical Committee (H-1-7001881) and conducted in accordance with the Helsinki Declaration (2002).

### Preoperative imaging and tracer preparation

**Fig. 1:**
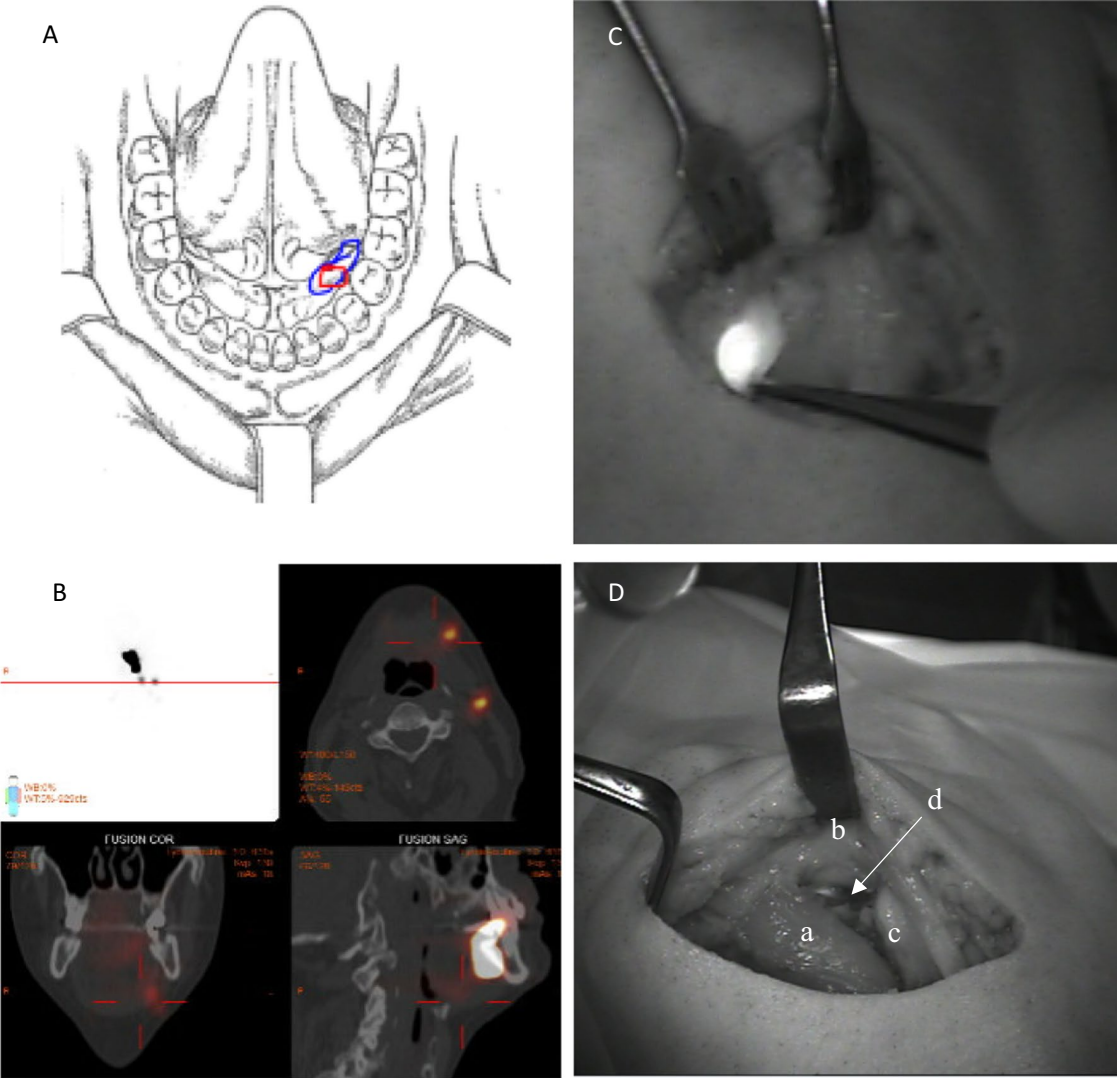
Optical imaging with the Fluobeam 800 system. An example of SNB staging of a cT1N0M0 lesion in the left FOM without midline involvement (**A**). SPECT/CT detected 2 SNs in the ipsilateral level 1b and 2a (**B**). Intraoperative optical imaging of the SN harvested in level 1b (**C**). The SN was directly visualized by optical imaging just below the edge of the mandible where navigation with the handheld gamma probe was impaired. The surgical field in the anterior level 1b after completed super-selective ND (**D**): Anterior belly of the digastric muscle (a), lower edge of the mandible (b), anterior portion of the submandibular gland (c), the location of the SN (d)

**Fig. 2:**
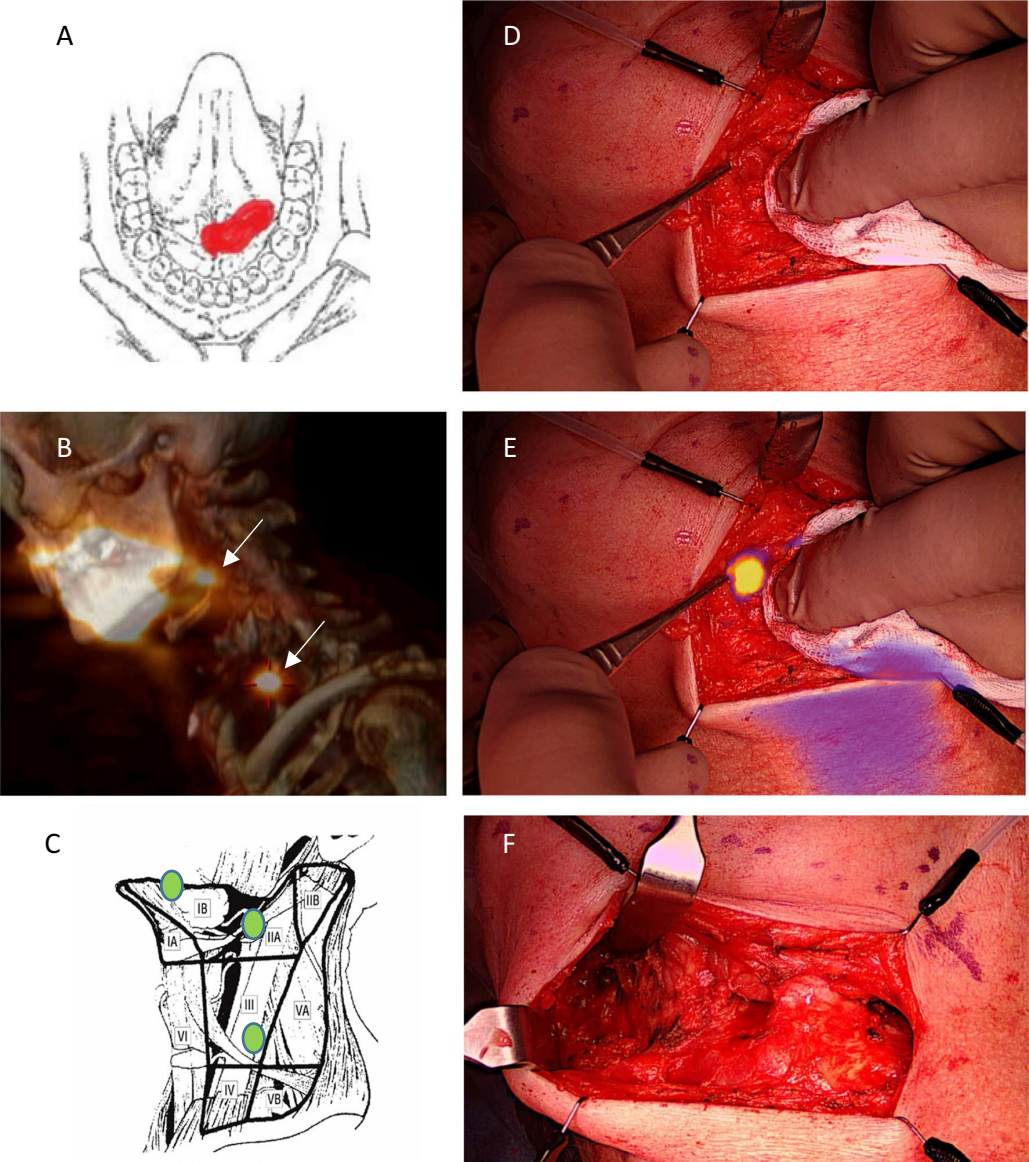
Optical imaging with the EleVision system. SNB staging of a cT2N0M0 lesion crossing the midline in the FOM (**A**). SPECT/CT showed two SNs in the ipsilateral level 2a and level 3 (**B**). Intraoperatively, a third SN was detected by optical imaging in the anterior level 1b that was not visible on SPECT/CT due to overshadowing form the primary injection side. The anatomical location of all three detected SNs in pictogram (**C**). Intraoperative imaging of level 1b close to the injection site in color (**D**), with merged ICG-based optical signal (**E**) and the surgical field after super-selective ND of the left anterior level 1b and level 1a

At the day of surgery, or the day before, the patients had a peri-tumoral submucosal injection of the bimodal tracer ICG:Tc99m:Nanocoll. The preparation of the tracer and the imaging protocol has previously been reported in detail [19]. Briefly, immediately after tracer injection by the surgeon, dynamic and static lymphoscintigraphy was performed, followed by a single-photon emission computed tomography/computed tomography (SPECT/CT). The preoperative imaging was reviewed by a nuclear medicine physician, and number and anatomical location of SNs were reported to the surgical team. A lymph node clearly identified on lymphoscintigraphy and SPECT/CT was reported as a SN. If no SN could be detected on initial imaging, a second scanning protocol was undertaken 90–120 min post-injection (see Figs. 1 and 2).

### Surgical procedure and intraoperative imaging

**Fig. 3:**
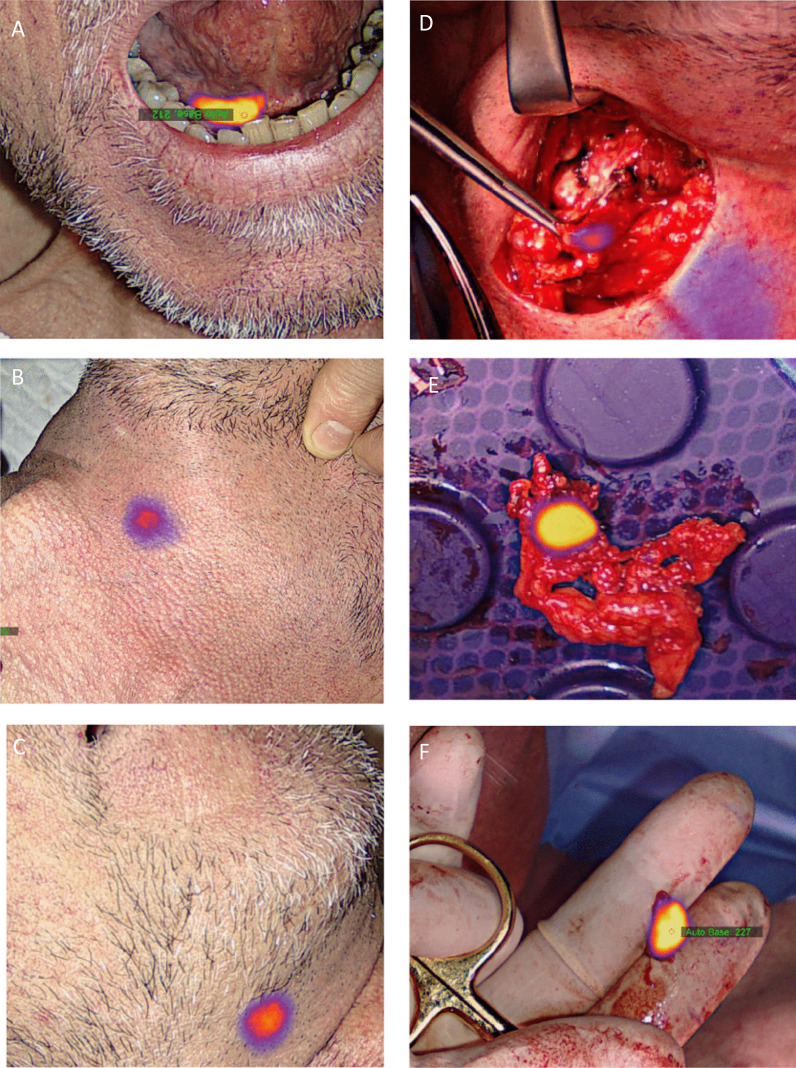
Transcutaneous optical SN detection with the EleVision system. Optical imaging of the injection site of a cT1N0M0 lesion in the left FOM (**A**). In the neck, bilateral drainage to level 1b was observed and both SNs could be visualized through the skin prior to surgery (**B**, **C**). Intraoperative optical of the right SN in level 1b (**D**) and ex vivo imaging of the left (**E**) and right (**F**) SN

The tumor ablation and the reconstruction were undertaken prior to SNB neck procedure. No free flaps were used in this series. For optical near-infrared fluorescence (NIRF) imaging, one of two available clinically approved optical camera systems, Fluobeam 800 (Fluoptics, France) or EleVision (Medtronics, USA) was used. The Fluobeam 800 generated NIRF imaging merged on video recording of the surgical field in gray scale. The camera head was handheld during the procedure, and imaging was presented on a screen on a chart for the surgical team. The EleVision recorded video in color-merged with the NIRF imaging presented in color or signal-intensity graded color. The camera head was mounted on a maneuverable surgical arm attached to chart with a screen. During imaging, surgical lights and headlights were turned away to diminish interference from near-infrared light from external light sources. Prior to incision, the SNs identified on preoperative imaging, were searched with a handheld gamma probe (Neo2000, Neoprope Corporation, USA), and the locations were marked on the skin. All patients had an upper submandibular neck incision to allow a super-selective ND of level 1 to be performed. If the tumor involved the midline, both neck sides were entered to access level 1. Depending on the location of the detected SNs on the preoperative imaging, second small incision in the lower part of the neck was needed in some cases to harvest SNs located in levels II–IV. To maintain oncological safety in the assessment of the possible diagnostic benefit of optical navigation for SN detection in level 1, it was decided, that all patients also had a super-selective ND of level 1 performed, instead of an optical exploration only. With this approach, possible subclinical nodal metastasis in level 1, not detected by optical guidance would be removed, and the histological examination of the neck dissection specimen served as reference for the accuracy of optical-guided SN detection. Navigation with a handheld gamma probe directed by the radioactive signal and NIRF imaging for the optical signal was performed in parallel to detect SNs. Specifically, for level 1, the surgical field was initially searched for possible SNs, not detected on preoperative imaging, with NIRF imaging, before the super-selective ND was performed. The anatomical borders of the super-selective ND in level 1b were the preglandular triangle defined by the lower edge of the mandible, the anterior border of the submandibular gland, the anterior belly of the digastric muscle and the mylohyoid muscle medially. The anatomical boundaries for level 1a were the midline space below the mylohyoid muscle between the anterior bellies of the digastric muscles and the hyoid bone inferiorly. Before closure, the surgical field was searched systematically with the gamma probe and the optical camera for any remnant signals. The dissected specimens from level 1 were also searched for radioactive or optical signals ex vivo. A lymph node was denoted a sentinel node if the radioactive count was at least 10% of the count of the hottest lymph node detected [9]. The individual gamma counts were registered, and optical images of all harvested SNs were stored. The exact anatomical location of all harvested SNs was recorded on a pictogram in a clinical report form. Contralateral drainage was defined as drainage to the opposite neck site from a tumor without midline involvement (see Fig. 3).

### Histological processing and follow-up

Harvested SNs were processed in a standard step-serial sectioning protocol based on H&E and cytokeratin antibody staining, as previously described [19]. Frozen sectioning technique was not applied. The level 1 ND specimens were examined for lymph nodes by routine histological analysis. Detected metastatic deposits were classified as macro-metastasis, micro-metastasis, or isolated tumor cells (ITC), respectively [20]. In case of a positive SNB, a completion ND was subsequently performed. All patients entered a 5-year surveillance program and were seen for regular clinical follow-up, every 3 month the first year, every 6 month the second and third year, and every 12 month the fourth and fifth year. Based on medical chart review, last follow-up was conducted on March 15, 2022.

### Data analysis

The software SAS Enterprise Version 9.4 (SAS Institute, Inc) was used for data analysis. Survival data was analyzed by use of the Kaplan–Meier method. Overall survival (OS) was defined as time from diagnosis until death of any course or censored at the last known follow-up. Recurrence-free survival (RFS) was defined as time from diagnosis until biopsy-validated recurrence or censored at the last known follow-up. For assessment of a possible association between depth of invasion (DOI) as a continuous variable and presence of a positive SN, a Student’s t test was applied. To access the diagnostic performance of SNB, the primary endpoint was false negative events defined as N-site recurrence in the initially SNB-negative group of patients. False negative rate (FNR, (FN/FN + TP), false omission rate (FOR, FN/FN + TN), and negative predictive value (NPV = 1-FOR) were calculated.

## Results

A total of 50 patients were enrolled in the study. Demographics and tumor characteristics are depicted in Table 1. A SN could be detected on preoperative imaging as well as intraoperatively in all patients. Hence, the rate of a successful SNB staging procedure was 100%. In 3 patients (6%), a second SPECT/CT was needed to identify a SN because the initial scan was without a detectable SN.

**Table 1:** Patient characteristics

Variable	*N*
No. Patients	50 (100%)
Sex	
Male	37 (74%)
Female	13 (26%)
Age (median, range)	66 (45–88) years
T-site	
FOM	43 (86%)
Inferior tongue	6 (12%)
Lower gum	0 (0%)
Buccal mucosa	1 (2%)
pT stage (AJCC8)	
T1	31 (62%)
T2	17 (34%)
T3	2 (4%)
Depth of invasion (mean, range)	3.3 (0.5–11) mm
Midline involvement	
Yes	23 (46%)
No	27 (54%)
Drainage pattern	
Ipsilateral	30 (60%)
Bilateral	20 (40%)
Contralateral	0 (0%)
Timing of tracer injection	
Same day	46 (92%)
Day before	4 (8%)
Tracer dose (mean, range)	
Same day	55.6 (51.0–63.6) MBq
Day before	111.8 (109.5–114.0) MBq
SNB status	
SN +	6 (12%)
SN–	44 (88%)

A total of 128 SNs were harvested with a mean of 2.6 SN pr. patient (range 1–5). The drainage patterns of the resected SNs are presented in Table 2. Of the lateralized tumors, 11% had bilateral drainage, whereas tumors with midline involvement demonstrated bilateral drainage in 74% of the cases. In 12 patients (24%), the neck level I was without a detectable SN on SPECT/CT, but intraoperatively a SN could be detected optically in this neck level anterior to the submandibular gland. All resected SNs were radioactive and fluorescent. In a few cases, a small lymph node was fluorescent, but without a relevant radioactive count above 10% of the count in the SN with the highest count and submitted for histology as non-SN. None of these fluorescent non-SNs contained metastasis after routine histological examination. In 22 patients (44%), 26 of the 128 harvested SNs could be detected only due to optical imaging, distributed with 20 nodes (77%) in level 1b and 6 nodes (23%) in level 1a. Five of the 26 nodes (5%) were located as part of a cluster of lymph nodes, and the majority of the nodes were located lateral to myeloid muscle at the posterior border of the anterior belly of the digastric muscle. In 2 cases, a SN was identified by optical imaging below the edge of the mandible and cranially to the upper edge of the submandibular gland, thus outside the extent of the planed super-selective ND. Of the 26 additional nodes, 24 were identified within the surgical field prior to the super-selective level 1 ND, whereas 2 lymph nodes were detected ex vivo when the resected specimen from the ND was searched with the optical camera. The median radioactive count, compared to the SN with the highest count, in the additional 26 SNs identified by optical imaging was 46% (range 10–100%). Two of the 26 SNs detected only due to NIR navigation, in 2 patients, contained metastasis and led to pN upstaging. However, it did not alone change a cN0 neck to a pN-positive neck. The two patients had a tumor on the inferior surface of the tongue and in the FOM, and in both cases, a SN with metastasis in level 2a was detected on preoperative imaging, and an additional SN with metastasis in level 1b was detected due to optical imaging. In the specimens from the super-selective level 1 ND in the SNB-negative group of patients, no metastatic deposits were detected. The DOI was significantly higher in the SN-positive group compared to the SN-negative group with a mean difference of 2.5 mm (95% CI 2.3.4.7, *p* = 0.02).

**Table 2:** Lymphatic drainage patterns

T-site	No. Neck sides	Neck level distribution								
		I	I + II	I + III	I + IV	I + II + III	II	II + III	III	IV
FOM	61	17 (28%)	11 (18%)	10 (16%)	1 (2%)	2 (3%)	11 (18%)	2 (3%)	5 (8%)	2 (3%)
Inferior Tongue	8	3 (38%)	1 (13%)	–	–	–	2 (25%)	2 (25%)	–	–
Buccal Mucosa	1	1	–	–	–	–	–	–	–	–

The table shows the combinations of drainage patterns based on T-site and SN location by neck level

The distribution stems from the 70 neck sides of the 50 patients staged by SNB of which 20 patients had bilateral SN drainage

Transcutaneous visualization of one or more SNs by optical imaging prior to the neck incision was possible in the majority of the patients. However, it was not recorded systematically as an endpoint in this study because data from a previous study found transcutaneous NIR-guided SN detection to be inconsistent between patients. Transcutaneous optical visualization was markedly improved if lights in the OR were dimed and if the skin was stretched by positioning of the neck. Also, imaging was improved if the camera head was brought closer to the skin (10–20 cm) and positioned perpendicular to the surface. Twenty-four of the cases were conducted with the Fluobeam system and 26 of the cases with the EleVision system. Both systems demonstrated a high sensitivity for the fluorescent signal from ICG retained in SNs intraoperatively, and in some cases, lymphatic vessels could be clearly visualized. In some cases, when a SN was resected and collecting lymphatic vessels were transected, localized spillage of fluorescent dye could be observed that could be misinterpreted as a possible additional SN located in the surgical field.

Of the 6 patients with a positive SNB, 4 patients had completion ND performed where no additional metastasis was found. A total of 8 positive SNs were retrieved, whereas 6 were located in level 1b and 2 were located in level 2a. The tumor deposits consisted of 1 macro-metastasis, 3 micro-metastasis, and 4 isolated tumor cells without signs of extra-nodal extension. In 2 patients, completion ND were waived due to patient frailty and decision of postoperative radiation therapy to T-site-related findings on histology, respectively. In total, 3 patients (6%) had postoperative radiation, whereas 1 patient had radiation upon recurrence. The median follow-up was 30 months (range 2–52 months). Two patients died from causes unrelated to the disease and 1 patient died from OSCC. No N-site recurrences were observed. The 2-year OS and RFS for the entire cohort were 96% and 96%, respectively. Based on the rate of N-site recurrences in the SNB-negative part of the cohort, the diagnostic accuracy of SNB neck staging in conjunction with super-selective level 1 ND was 100%, and the false negative rate and false omission rate were 0%.

## Discussion

The results from this study underline the challenge of monomodal radiocolloid-based SNB neck staging of tumors in the anterior oral cavity, which has been described in previous reports as a lower diagnostic accuracy compared to other sub-sites in the oral cavity [7, 8, 21, 22]. The current study shows a substantial risk of undetected SNs in level I in close vicinity to the injection site on preoperative scintigraphy and SPECT/CT must likely caused by the shine-through effect. Similar, in a study of 40 patients with FOM tumors, managed with SNB in conjunction with a super-selective level 1 ND, 50% of the harvested SNs in level 1 were not detected on the preoperative SPECT/CT [10]. However, this current study also indicates, that when SNs in level 1 are systematically searched and resected, aided by additional optical imaging and super-selective level 1 dissection, an excellent accuracy of staging a cN0 neck can be achieved. Hence, SNB staging of FOM tumors is not to be advised against but adapted surgical technique and technology should be adopted to manage the risk of subclinical nodal disease in level 1.

Importantly, the primary purpose to explore the level 1, is to detect a possible SN to obtain accurate nodal staging by step-serial sectioning technique and not just clear the level as part of a SND. Direct optical-guided exploration of level 1 to detect possible SNs without subsequent resection of the fibrofatty tissue in anterior level 1b is technically feasible. Such strategy would adhere to the concept of minimal invasiveness of SNB which is one of the major advantages of this procedure. However, in this study, primary exploration followed by a super-selective ND was preferred to maintain high oncological safety in a protocolized setting to evaluate the diagnostic performance of optical SN detection and to collect additional clinical experience with the combined radio- and optical-guided approach.

Based on the results from this study, our current clinical practice is to perform combined optical- and radio-guided SNB with transcutaneous and intraoperative optical imaging in level 1 without a super-selective ND.

The main advantage of the additional use of optical imaging is that it allows direct real-time visual detection of lymph nodes in high spatial resolution. Lymph nodes imbedded in tissue were also detectable due to relatively high penetration of NIR photons in the range of 1–2 cm. In addition, optical-guided SN detection works independently from the radioactive signal and thereby seems to circumvent the challenge of radiocolloid-mediated shine-through from the injection site in level 1. Also, in this study, SNs located just below the mandible but outside the extent of the performed super-selective ND were detected optically, which exemplifies both the variation in SN drainage patterns and the ability of this technology to detect SNs in less common locations. It could be argued, that a primary optical exploration of level 1 followed by a completion neck dissection in a second procedure, due to a positive SN, would carry an increased risk of damage to the marginal branch of the facial nerve due to scare tissue. In our current experience with the use of optical-guided SN detection, we have not observed issues with nerve damage, but the rate of possible complications should be studied in future clinical studies. Transdermal optical detection of SNs in the neck region appears to be a helpful tool for fast and direct SN localization but deeply located SNs may not be visible [23]. In breast cancer, transcutaneous optical technique based on ICG for SN detection has been reported [24].

Optical-guided SNB based on ICG has been explored in several types of cancers with reported high SN detection rates and a superior diagnostic performance when compared to blue dye, which previously has been used extensively as a visible lymphatic tracer [25, 26]. In head and neck malignancies, the use of ICG alone [11, 23, 27–29] or ICG non-covalently bound to radio-colloids [12, 13, 16, 17] has been investigated. Due to a low molecular size, ICG alone has a fast lymphatic drainage kinetics and limited retention in the first echelon node and therefore uptake in downstream non-SN is a potential challenge [30]. By coupling of ICG to a colloid to create a tracer complex, retention in the SN is increased [31]. As an alternative, a first injection with “hot” Tc99m:Nanocoll for preoperative imaging and a second “cold” injection prior to surgery and in general anesthesia with ICG:Nanocoll has been suggested, to allow a more flexible timing of imaging and surgery [14]. Combined radio- and optical guidance provides complementary characteristics for SN detection, and preoperative knowledge of the drainage pattern and anatomical relations in the neck is of key importance of a successful SNB procedure. In some cases, in this study, an optical signal in lymph nodes that proved without a relevant radioactive count was seen, probably due presence of a low amount of tracer complex. Also, compared to detection of gamma photons, very low concentrations of ICG in the surgical field are required to produce a strong optical signal. Therefore, exploration of individual optical distinct signals within the surgical field was sometimes needed to determine if the signal represented a lymph node. Generally, a learning curve is to be anticipated when combined radio- and optical-guided SNB is introduced.

The SNB positivity rate was only 12% in this present study with is markedly lower compared to data form our own and other published series with a positivity rate in the range 20–30% of cN0 in early OSCC [8, 32]. This finding may be explained by improved early detection and referral of oral cancer lesions, but we have limited data to support this [33]. Because the SNB positivity rate also influences on the calculation of the diagnostic performance of SNB, more data from patients with higher risk of subclinical nodal disease are needed to evaluate possible added effects, for example in terms of nodal upstaging, only due to optical guidance.

The drainage patterns described in this cohort support the well-documented involvement of primarily levels 1–3 in early-stage OSCC, with only 3% of harvested SNs outside this region. However, the data also show a substantial variation in the combinations of involved neck levels harboring SNs. Importantly, 40% of the patients had simultaneously combined lymphatic drainage to both level 1 and another neck level, which demonstrates the need to carefully explore the level 1, also if preoperative imaging only suggests drainage outside level 1. These observations very likely explain the mechanism of nodal recurrences in SNB-negative patients in previously reported series where nodal metastases were present upstream from harvested SNs in level 2 or 3 but remained undetected [34].

Apart from optical imaging, other technologies have been explored to overcome the challenge of shine-through to accurately detect SNs in level 1 like portable SPECT or gamma cameras for intraoperative and postoperative imaging [35, 36]. Also, novel tracers with enhanced targeted retention in SN, radio-opaque CT-based lymphogram imaging or more optimal radio-metals have been reported [37–39]. Most recently, SNB based on MR imaging and ferro-tracer particles that permits both preoperative imaging and intraoperative spatial detection of SNs has been introduced with promising perspectives of a non-nuclear SNB approach [40].

## Conclusion

Combined optical and radio-guided SNB in conjunction with a super-selective level 1 ND accurately staged the cN0 neck in patients with OSCC sub-sites in the anterior oral cavity with a false omission rate of 0%. Optical imaging enabled direct intraoperative real-time visualization of SNs, and in 46% of patients, additional SNs in level 1 were detected due to this modality. Exploration of level 1 irrespective of findings on preoperative radio-based imaging is recommended for tumors in FOM and other locations that carry risk of shine-through.

## Data Availability

Access to data is available on reasonable request to the corresponding author.

## Abbreviations

SNB: Sentinel node biopsy
OSCC: Oral squamous cell carcinoma
FOM: Floor of the mouth
ICG: Indocyanine Green
ND: Neck dissection
SPECT/CT: Single-photon emission computed tomography/computed tomography
NIRF: Near-infrared fluorescence
OS: Overall survival
RFS: Recurrence-free survival
DOI: Depth of invasion
